# Mechanical and Hydric Stress Effects on Maize Root System Development at Different Soil Compaction Levels

**DOI:** 10.3389/fpls.2019.01358

**Published:** 2019-10-29

**Authors:** Moacir Tuzzin de Moraes, Henrique Debiasi, Julio Cezar Franchini, João de Andrade Bonetti, Renato Levien, Andrea Schnepf, Daniel Leitner

**Affiliations:** ^1^Department of Agronomic Science, Federal University of Technology-Paraná campus Francisco Beltrão, Francisco Beltrão, Brazil; ^2^Department of Soil and Crop Management, Embrapa Soybean, Londrina, Brazil; ^3^Department of Agronomy, State University of Maringa, Maringa, Brazil; ^4^Department of Soil Science, Federal University of Rio Grande do Sul, Porto Alegre, Brazil; ^5^Forschungszentrum Juelich GmbH, Institute of Bio- and Geosciences, IBG-3: Agrosphere, Juelich, Germany; ^6^Services in Computational Science, Simulationswerkstatt, Leonding, Austria

**Keywords:** root growth modeling, drought stress, soil strength, soil physical limitation, *Zea mays*

## Abstract

Soil mechanical resistance, aeration, and water availability directly affect plant root growth. The objective of this work was to identify the contribution of mechanical and hydric stresses on maize root elongation, by modeling root growth while taking the dynamics of these stresses in an Oxisol into consideration. The maize crop was cultivated under four compaction levels (soil chiseling, no-tillage system, areas trafficked by a tractor, and trafficked by a harvester), and we present a new model, which allows to distinguish between mechanical and hydric stresses. Root length density profiles, soil bulk density, and soil water retention curves were determined for four compaction levels up to 50 cm in depth. Furthermore, grain yield and shoot biomass of maize were quantified. The new model described the mechanical and hydric stresses during maize growth with field data for the first time in maize crop. Simulations of root length density in 1D and 2D showed adequate agreement with the values measured under field conditions. Simulation makes it possible to identify the interaction between the soil physical conditions and maize root growth. Compared to the no-tillage system, grain yield was reduced due to compaction caused by harvester traffic and by soil chiseling. The root growth was reduced by the occurrence of mechanical and hydric stresses during the crop cycle, the principal stresses were mechanical in origin for areas with agricultural traffic, and water based in areas with soil chiseling. Including mechanical and hydric stresses in root growth models can help to predict future scenarios, and coupling soil biophysical models with weather, soil, and crop responses will help to improve agricultural management.

## Introduction

Soil compaction is considered the main cause of the physical degradation of agricultural soils (Nawaz et al., 2013). However, soil compaction alone is not always the problem, and therefore a quantification how it affects crop yield and root growth is of high relevance. The effect of soil compaction has led to many debates, especially regarding the establishment of critical limits to plant growth (Moraes et al., 2014; De Jong van Lier and Gubiani, 2015). If there is no chemical or biological limitation, plant growth is mainly directly affected by four physical factors in soils, which are temperature, aeration, resistance, and water content (Letey, 1985). Moreover, it is indirectly affected by several soil characteristics, for example, texture, aggregate structure, and pore size and distribution (Letey, 1985).

Mathematical modeling has become an important tool for describing the functionality of biophysical processes including soil–root interactions, such as water absorption and root growth (Dupuy et al., 2010). The physical conditions of the soil that influence plant growth are dynamic in time and space, and an improvement in the techniques used for the understanding of these conditions is necessary. The use of eco-hydrological models for root growth is, therefore, an important way to increase the understanding of dynamics of soil–plant–atmosphere interactions (Tron et al., 2015). There are many different root system models that can be divided into pure root growth models (Hartmann et al., 2017), such with a focus only on root system’s topology (Berntson, 1997; Pagès et al., 2004) or more mechanistic models (Javaux et al., 2008; Leitner et al., 2010b; Moraes et al., 2018a). The decision to use a specific soil–root model should be related to input data (Bengough, 1997; Javaux et al., 2008; Schnepf et al., 2018), computer power availability (Dupuy et al., 2010), and expected output (Dunbabin et al., 2013). However, there are still very few models describing soil–plant interactions, therefore between the physical (*e.g.*, soil water flow, soil mechanics, gas flow, or solute transport) and biological (*e.g.*, water uptake/release, root system architecture, or shoot and root growth) processes to predict root growth (Vereecken et al., 2016).

The rate of maize root elongation is strongly correlated with soil mechanical resistance to penetration and with the matric potential (Bengough et al., 2011). No functional–structural model of root growth and root water uptake currently addresses the dynamics of those stresses and their effect on maize root elongation during the crop development cycle (Leitner et al., 2010a; Dunbabin et al., 2013; Leitner et al., 2014). In Moraes et al. (2018b), we presented a new model of hydric and mechanical stress effects on root elongation. The model consists of two modules, an extended version of the RootBox model (Leitner et al., 2010a) that reduces root elongation rate due to mechanical and hydric stresses, and a 1D solution of the Richards equation including root water uptake. The model was evaluated with field data for soybean, and we could show that explicitly distinguishing mechanical and hydric stresses induced by dynamically changing soil physical conditions is fundamental for advancing the knowledge of soil–root relationships in soil management. This is because during the crop development cycle, plants undergo a series of phenological changes that affect the transfer of mass and energy in the soil–plant–atmosphere system (De Jong van Lier and Gubiani, 2015).

We hypothesized that maize root growth can be simulated with RootBox model as a function of soil physical condition (mechanical impedance, hypoxia, and water stress) in various compaction levels of an Oxisol. The objective of this work was to evaluate whether this new root growth model can be used to interpret field data of maize grown in a Rhodic Eutrudox in Brazil at different levels of soil compaction. In particular, we aimed to identify the individual contributions of mechanical and hydric stresses to root elongation reduction and thus help to improve soil management at this site. The measurements available from this field site, including the stress reduction function (*i.e.*, mechanical and hydric stresses to reduce root elongation), serve as a reference against which our model is evaluated. As field data for benchmarking root growth models are scarce, we make our results an example of way to serve for benchmarking of other root growth models as well.

## Materials and Methods

### History and Characterization of the Area

The experiment was set up in an area located at Embrapa Soybean, in Londrina (latitude 23°12′S; longitude 51°11′W; 585 m in altitude), in the southern Brazilian state of Paraná. The climate, according to the Koppen classification, is humid subtropical (cfa), with a mean annual temperature of 21°C and precipitation of 1,650 mm (Alvares et al., 2013). The soil of the experimental area was classified as an Oxisol [Latossolo Vermelho Distroférrico, in the Brazilian soil classification (EMBRAPA, 2006); and Rhodic Eutrudox, in the USA soil classification (Soil Survey Staff, 2014)] with 784 g kg-^1^ of clay, 145 g kg-^1^ of silt, and 71 g kg-^1^ of sand. The mean particle density of the 0–30 cm layer is 2.96 Mg m^−3^, and the land slope is 0.03 m m^−1^.

The area was cultivated with coffee (*Coffea arabica* L.) for approximately 40 years, with no-tillage system introduced in 1991. From 1991 to 2009, crops were grown in rotation systems, with soybean (*Glycine max*) or maize (*Zea mays*) in the summer (seeding in October), and wheat (*Triticum aestivum*) or black oats (*Avena strigosa*) in the winter (seeding in April). During the period from 2010 to 2012, *Urochloa ruziziensis* was cultivated in the summer and winter as a cover crop (without grazing). In the year 2013, soil compaction levels were applied to start this experiment (February 2013), and *U. ruziziensis* was desiccated 90 days before the initiation of the experiment (*i.e.*, December of 2012). In the winter crop season of 2013 (May to June of 2013), the area was cultivated with wheat with a row spacing of 17 cm, and in the summer crop season (October to February of 2013/2014) the soybean was spaced 45 cm apart more details about winter (2013) and summer (2013/14) crop season can be obtained in Moraes (2017).The maize crop (cultivar AG9010) was sown in second growth season (February to August of 2014), on February 27, 2014, using a seed drill (Semeato, model SHM 11/13), with three rows spaced 90 cm apart (seeding description below).

### Experimental Design, Treatments, and Cultivation

The treatments were setup in February 2013, and the experimental design was a randomized block with four treatments and three replicates, in plots 5 m wide and 15 m long. The treatments consisted of four levels of compaction: (1) MTC: minimum tillage system with soil chiseling; (2) NT: no-tillage system; (3) NTC4: no-tillage system with four tractor passes; (4) NTC8: no-tillage system with eight harvester passes.

Soil chiseling was performed when the soil had a friable consistency (gravimetric moisture of 0.29 kg kg^−1^). For the chiseling, a chisel plow was used (KLR ES5CR model) with rollers and five shanks spaced 35 cm apart (with a shovel tip of 8 cm), working at a depth of 25 cm. Compaction in the NTC4 treatment was performed through the traffic of a CBT 4×2 TDA tractor, model 8060, equipped with a loader on the front axle. The total mass of the tractor was 7.2 Mg (4.3 Mg in rear axle). The tires used on the front axle were Goodyear 14.9-24-R1, while Goodyear 18.4-34-R1 tires were fitted at the rear. The tractor rear axle was ballasted with masses of liquid into the tire (tire volume filled with 75% of water and 25% of air) and solids counterweights (cast iron weights) on wheels. The tire–soil contact pressure on the rear axle was estimated to be 180 kPa, through the procedure proposed by O’Sullivan et al. (1999).

The compaction in the NTC8 treatment was conducted through the traffic of a John Deere self-propelled harvester, model SLC-6200, equipped with a maize harvesting platform (weight of 1.2 Mg), with the grain tank empty, so the total weight of the harvester was 9.5 Mg (6 Mg on the front axle). The harvester was equipped with diagonal-ply tires (model 18.4-30 R1; Pirelli) on the front axle inflated to a pressure of 180 kPa. The tires on the rear axle were Pirelli, model 9.00-16 F2 10PR, diagonal-ply tires (inflated to a pressure of 410 kPa). The tire–soil contact pressure on the front axle was estimated to be 220 kPa, through the procedure proposed by O’Sullivan et al. (1999). Both traffic treatments, with tractor and with harvester, were carried out when the soil had a moisture level equivalent to that of field capacity (0.34 kg kg^−1^).

The maize crop was sown (cultivar AG9010, sowing February 27, 2014) to a population of 80,000 plants ha^−1^, with a 90-cm spacing between rows, and 5 cm deep. Fertilization of the crop was performed in the sowing furrow at a depth of 10 cm, with NPK 08-20-20, applied at 300 kg ha^−1^. A broadcast application of nitrogen fertilizer (ammonium sulfate) was made at a rate of 80 kg ha^−1^ of nitrogen during the V6 maize growth stage.

### Sampling and Soil Water Retention Curve

Undisturbed soil samples were collected in the period between soybean harvest and maize sowing (*i.e.*, February 26, 2014) from the 0–10, 10–20, 20–30, 30–40, and 40–50 cm layers, at three locations along the row and interrow, with 12 replications for each of the four compaction levels, giving 240 samples in total. For the determination of the soil water retention curve, the samples in the first three layers were saturated and submitted to matric potentials of −3 and −6 kPa on a tension table, and −10, −33, −100, and −500 kPa in Richards pressure chambers. After equilibrium was attained at each matric potential, the samples were then weighed, and the soil penetration resistance was determined using a static bench penetrometer (Model: MA 933 Marconi). Furthermore, the soil core samples were oven dried at 105°C for 48 h, to quantify the water content and soil bulk density. Degree of compaction was calculated as the ratio between the bulk density and the maximum bulk density from proctor test (1.53 g cm^−3^) (Moraes et al., 2012).

Soil water content at −1,000 and −1,500 kPa (permanent wilting point) was estimated through readings of the soil water potential on a psychrometer (water potential meter, model WP4-C). Soil samples were air dried and sieved through a 2-mm sieve in the laboratory. Soil samples from each treatment were wetted and homogenized in a plastic container. The container was kept open for gradual soil water evaporation at ambient temperature (20–25°C). Eight samples (from each treatment) of approximately 5 g were withdrawn and transferred to the WP4 sample cup, which was then sealed with a lid and left on the WP4 around 5 min to equilibrate sample temperatures to the chamber temperature (Campbell et al., 2007). Soil water potential at the range of −700 to −2,500 kPa was measured on soil samples using a WP4 calibrated with 0.5 M KCl solution (Campbell et al., 2007; Gubiani et al., 2013). The power function was fitted to estimate the soil water content as function of water potential (Campbell et al., 2007). Volumetric soil water content at those water potentials (−1,000 and −1,500 kPa) was obtained by multiplication of gravimetric water content and bulk density.

We describe the soil water retention curve following van Genuchten (1980). Plant-available water capacity (mm) was estimated for the 0–50 cm depth from the difference between water content at −10 kPa and that water content at −1,500 kPa by the product between water available and the layer thickness (mm). The frequency of the distribution and accumulation of the pores was determined from the first derivative of the van Genuchten equation, and the equivalent pore diameter (EPD) was estimated by EPD=300/*h* (where *h* is the matric potential in kPa, and EPD is expressed in µm) (Hillel, 2004).

### Grain and Biomass Productivity

The grain yield was evaluated by mechanical harvesting, on August 6, 2014, along four central rows 13 m in length, totaling an area of 46.8 m^2^. The grains were threshed, weighed, and their mass corrected for 13% moisture. Shoot biomass was determined through the collection of two 1-m (1.8 m^2^) rows, which were dried in forced air circulation greenhouses at 60°C until achieving a constant weight.

### Sampling and Analysis of the Root System

Root system sampling was performed on June 18, 2014 (*i.e.*, 111 days after sowing), following the monolith methods considering the simple spade methods described in Böhm (1979). In each field plot, a trench 90 cm wide, 8 cm thick, and 50 cm deep was opened and sampled. In the trenches, soil–root monoliths were collected transversely to the maize rows (Böhm, 1979), which were divided into five layers each 10 cm deep (0–10, 10–20, 20–30, 30–40, and 40–50 cm), and three positions of 30 cm (row and interrow), one in the row and two in the interrows of the plants, totaling 180 small monoliths. These soil blocks were taken from the trench wall using a knife and a spatula to sample the exact soil volume for each small monoliths.

The roots were separated from the soil by washing under running water, using sieves with a mesh diameter of 0.5 mm. After root washing, approximately 10% of the roots were sampled for digitization using a scanner (Delta-T Scan). After digitization, root length was determined by image processing using the program for fiber and root analysis, Safira 2.0 (Jorge and Silva, 2010). The weight of the scanned root sample and its respective root length were related to the total weight of the sample to obtain the total root length in each small monolith.

The root length density (cm cm^−3^) of the maize root system was determined by the ratio of the root length (cm) and the soil volume sampled in the small monoliths (30×10×8 cm^3^). The dry root biomass was determined by drying in a forced air circulation greenhouse at 60°C until a constant weight, with the root biomass density results expressed as a function of the soil surface area (g m^−2^ per 10 cm depth).

### Climatic Data

Climatic data regarding the development period of the maize were collected at the Embrapa Soybean meteorological station, located near the experimental area. Thus, data were collected daily for solar radiation, temperature, relative humidity, wind speed, and precipitation during the development of the crop. The reference evapotranspiration was calculated from the meteorological data using the Pennan–Monteith equation (Allen et al., 1998).

### The Root Model: Modeling Approach for Maize Root Growth

A root growth model from Moraes et al. (2018a) previous used for soybean root growth was applied to maize root growth. Thus, the root growth model is composed by (1) the root architecture model RootBox, (2) a soil–water redistribution model using Richards’ equation and a water uptake function, (3) a soil-strength function that relates soil strength to soil water status, and (4) a root-stress function to define root elongation rate as limited by soil physical conditions. The RootBox model was described previously (Leitner et al., 2010a), and that model including soil physical conditions for root growth was already described for soybean in Moraes et al. (2018a). Thus, the main changes of the model components are described in the following sections, together with the input parameters used in a series of comparison with compaction levels from field tests.

### Root Growth Modeling

Root growth was modeled in the MatLab^®^ programming and the root growth code was available online in **Supplementary Material** the effects of soil physical limitations on root elongation (stress reduction function) proposed by Moraes (2017) were incorporated into the RootBox model of root architecture in 3D of Leitner et al. (2010a). Thus, the 3D root growth model was coupled to a 1D soil water flow model using equations proposed by Van Dam and Feddes (2000) and to the root water absorption model proposed by de Jong Van Lier et al. (2008). The 1D hydrological model of soil water flow numerically solves the Richards equation and can simulate water flow in the soil–plant–atmosphere continuum (van Dam and Feddes, 2000). The equations of the model, and its coupling and implementation were described in Moraes et al. (2018b). In addition, we performed a baseline scenario where the root system (*i.e.*, root length density) was modeled without considering the stress reduction function as a guide to quantify the effect of soil physical limitation from soil compaction levels.

### Soil Penetration Resistance and Soil Strength Function

Soil penetration resistance, a measure of soil strength, varies greatly with soil water status, and was modeled as a function of soil water content and bulk density using a non-linear model (Eq. 1) (Busscher, 1990). Soil penetration resistance was measured with a penetrometer in soil cores after hydrostatic equilibrium. That penetrometer consisted of a metal rod with a cone at the end, with a semi-angle of 30°, 4 mm diameter, and with a base area of 0.1256 cm^2^ connected to a load cell with a nominal capacity of 20 kgf. The penetration rate was 20 mm min^−1^, so that for each sample, 120 readings were performed to a depth of 40 mm. The soil penetration resistance was calculated as the average of the readings from 5 mm to 40 mm soil depth for each core sample. Data of soil penetration resistance, water content, and bulk density were used for characterization of soil strength fitting the constants (*a*, *b*, and *c*) using Eq. (1).

(1) $$Q_{p}=a\gamma^{b}\theta^{c}$$

where *Q_p_* (MPa) is the soil penetration resistance; γ (g cm^−3^) is the dry bulk density; θ (cm^3^ cm^−3^) is volumetric soil water content; and *a*, *b*, and *c* are empirical parameters.

**Figure 1:**
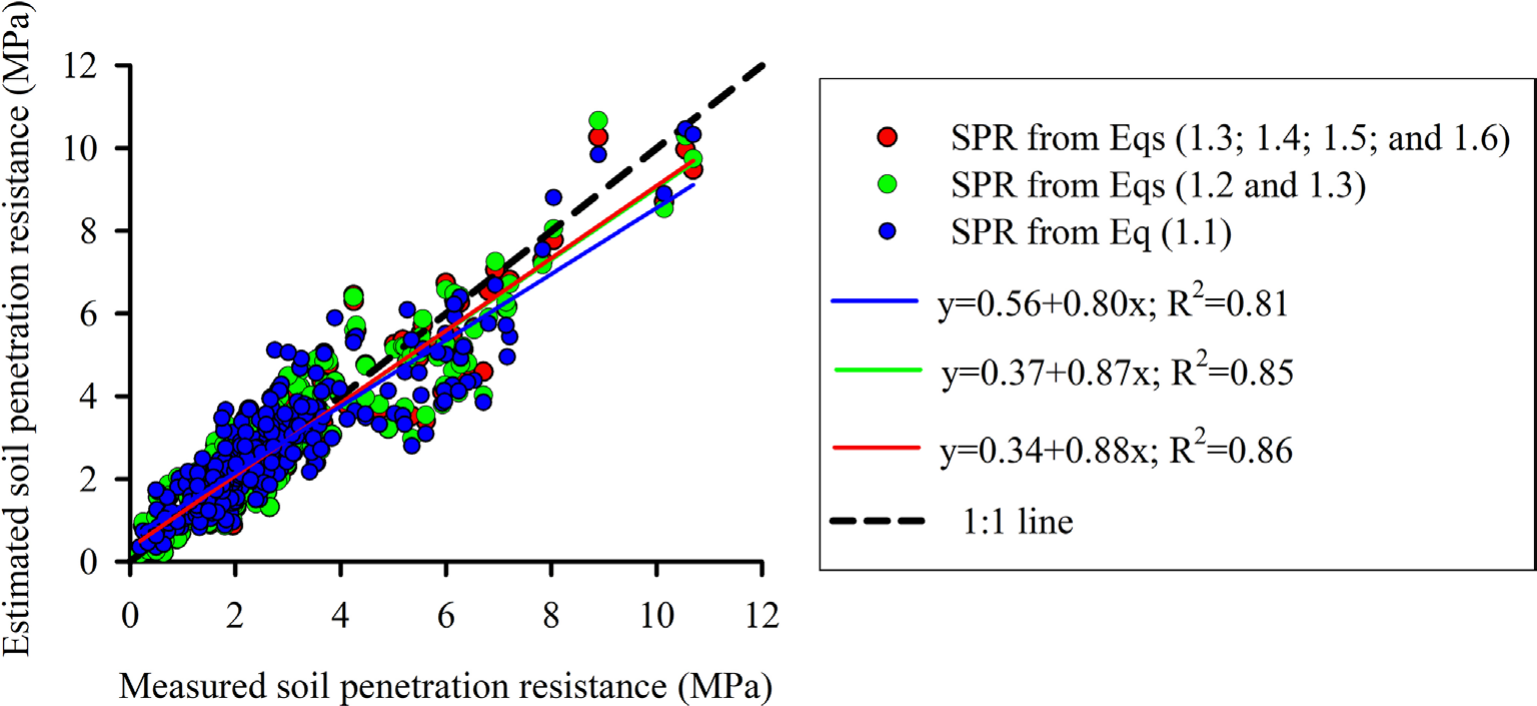
Measured *vs*. calculated values of soil penetration resistance (SPR) curve fitted with parameters of Busscher equation from **Table 1** for a Rhodic Eutrudox, very clayed. Dashed line represents a one-to-one relationship.

The soil penetration resistance curve used for soil characterization was fitted using three strategies: (1) parameter fitting using data from all treatments (MTC, NT, NTC4, and NTC8); (2) parameter fitting using data from no-tillage and soil traffic (NT, NTC4 and NTC8); (3) parameter fitting for each treatment (**Table 1**). Estimated soil penetration resistance values agreed with the measured values (**Figure 1**). In addition, the soil penetration resistance values estimated with three strategies of parameter fitting—(1) SPR for all treatments (Eq. 1.1), (2) SPR for tillage systems (Eqs. 1.2 and 1.3), and (3) SPR for each treatment (Eqs. 1.3, 1.4, 1.5, and 1.6)—have shown similar results (**Figure 1**). Thus, in this study, we used Busscher equation (Eq. 1.1) with soil strength parameters fitted including all compaction levels (*i.e.*, *a*=0.00587, *b*=8.0772, and *c*=−4.65).

**Table 1:** Empirical parameters fitted to the models of soil penetration resistance in soil compaction levels in an Oxisol.

Compaction level	SPR model^1^	*R*^2^	Equation
All treatments	*Q_p_*=0.00587 BD^8.0772^θ^−4.65^	0.96*	(1.1)
All NTs	*Q_p_*=0.00562 BD^6.6124^θ^−5.2170^	0.93*	(1.2)
MTC	*Q_p_*=0.00669 BD^9.1823^θ^−4.1110^	0.92*	(1.3)
NT	*Q_p_*=0.00341 BD^6.7933^ θ^−5.6626^	0.93*	(1.4)
NTC4	*Q_p_*=0.00661 BD^6.5249^ θ^−5.0519^	0.95*	(1.5)
NTC8	*Q_p_*=0.00674 BD^4.7799^ θ^−5.7517^	0.94*	(1.6)

Means followed by the same letter did not differ at the 5% level of probability by Tukey test. All treatments are all compaction level (MTC: minimum tillage with chiseling; NT: no-till system; NTC4: no-tillage with four passes of a tractor; NTC8: no-tillage with eight passes of a harvester) data used to fit the Busscher equation. All NTs are soil physical data from NT, NTC4, and NTC8.

^1^Soil penetration resistance model: Q_p_ = a×γ^b^×θ^c^; BD: bulk density (Mg m^−3^); θ: soil water content (m^3^ m^−3^); R^2^ = [1−(SQerror/SQmodel)]; *significant at 5% level of probability by F-test.

### Stress Reduction Function Model: Root Elongation as a Function of Soil Physical Stresses

We describe root elongation is a function of both soil strength and matric potential. We further assume that the combined effect of the two stresses (mechanical and hydric) is multiplicative for each time and depth, *i.e.*, the decrease elongation rate can be described by Eqs. (2)–(3) (Moraes et al., 2018a),

(2) $$\mathrm{RE}{\left(Q_{p},\;h\right)}_{t,z}=\mathrm{srf}{\left(Q_{p},h\right)}_{t,z}\text{R}{\text{E}}_{\max}$$

(3) $$\mathrm{srf}{\left(Q_{p},\;h\right)}_{t,z}=\alpha {\left(Q_{p}\right)}_{t,z}\alpha {\left(h\right)}_{t,z}$$

where *srf* (*Q_p_*, *h*)_t,z_ is the total stress reduction function for root elongation due to mechanical (*Q_p_*) and hydric (*h*) stresses in each time (*t*) and depth (*z*), α(*Q_p_*) is the stress reduction function by soil strength and is given by Eq. (4) for a soil with continuous macropores (Moraes et al., 2018a), α(*h*) is the stress reduction function by matric potential (water and aeration stress) given by Eq. (5), *t* is the time (day), *z* is the depth (cm), RE_max_ is the root elongation maximal possible without restrictions (cm day^−1^), and RE is the root elongation (cm day^−1^).

### Root Elongation in Relation to Soil Strength

Root elongation reduction due to soil strength–induced stress in layer *z*, on day *t*, is given by Eq. (4) following the recommendation of Moraes et al. (2018b) for a soil with continuous pores in the soil profile:

(4) $$\alpha {\left(Q_{p}\right)}_{t,z}=\exp(-0.30Q_{p})$$

### Root Elongation Under Water Stress and Poor Aeration

Under non-optimal hydric conditions, *i.e.*, either too dry (water deficit) or too wet (poor aeration), root elongation is reduced by means of the stress reduction factor α(*h*), ranging from 1 (maximum root elongation) to zero (no growth) (Moraes et al., 2018a). The relationship between root elongation rate and matric potential is described by Eq. (5) in terms of five stages: (1) no root growth due to anoxia condition |*h*| < |*h*_1_|; (2) root elongation rate was increased linearly from |*h*_1_| to |*h*_2_| due to increment of soil aeration; (3) no hydric stress of root elongation from |*h*_2_| to |*h*_3_|; (4) root elongation rate was reduced linearly due to water stress from |*h*_3_| to |*h*_4_|; (5) no root growth due to water stress. The *h*_1_ (−0.1 kPa) was defined at the wet end and represents the start of water drainage and increase of soil aeration (and oxygen concentration) necessary for root growth (Dresbøll et al., 2013). The *h*_2_ (−6 kPa) and *h*_3_ (−10 kPa) are the values close to field capacity (Iijima and Kato, 2007), when there is no water stress and thus root elongation rate is at its maximum. The *h*_3_ (−1,000 kPa) was defined as the limit of maximum growth due to turgor pressure in the expanding cells of the root elongation zone (Bengough et al., 2011).

(5) $$\alpha {\left(h\right)}_{t,z}=\{\begin{array}{ccc} 0 & \text{ if} & \left|h|\leq |h_{1}\right|\\ \frac{\left(|h_{1}|-|h|\right)}{\left(|h_{1}|-|h_{2}|\right)} & \text{ if} & \;|h_{1}|<|h|\leq |h_{2}|\\ 1 & \text{ if} & \;|h_{2}|<|h|\leq |h_{3}|\\ \frac{\left(|h_{4}|-|h|\right)}{\left(|h_{4}|-|h_{3}|\right)} & \text{ if} & \;|h_{3}|<|h|\leq |h_{4}|\\ 0 & \text{ if} & \;|h|>|h_{4}| \end{array}$$

where α(*h*) is the stress reduction factor of root elongation due pressure head; |*h*| is the module of pressure head; and *h*_1_, *h*_2_, *h*_3_, and *h*_4_ are the limits of pressures head for root elongation. Root elongation below |*h*_1_| (critical respiratory oxygen pressure, with |*h*_1_| approaching to saturation (1 cm) (Saglio et al., 1984)) and above |*h*_4_| (maximum growth pressure, with |*h*_4_| approaching 1,000 kPa (Bengough et al., 2011)) is set equal to zero. Between |*h*_2_| and |*h*_3_| (reduction point |*h*_2_| is 6 kPa, and |*h*_3_| is 10 kPa) root elongation is maximal. Between |*h*_1_| and |*h*_2_| and between |*h*_3_| and |*h*_4_|, a linear variation is assumed.

### Model Inputs and Outputs

The input parameters of the model were the soil characteristics (soil water retention curve, soil penetration resistance curve, saturated hydraulic conductivity, and soil bulk density) (**Tables 1** and **2**), climate characteristics (evaporation and transpiration potential, temperature, air humidity, precipitation, and irrigation), crop root characteristics (length of apical and basal zone, spacing between branches, number of branches, and root insertion angle), type of tropism, period of growth and physical limitations of resistance, and matric potential for root elongation. The input parameters for the maize root system architecture were adapted from Leitner et al. (2010b) and calibrated for this experiment (**Table 3**). The model calibration was performed by adjustment between simulated and measured root length density values using the parameters described at the item performance evaluation of the maize root growth simulation model.

**Table 2:** Van Genuchten’s parameter, hydraulic conductivity, bulk density, and degree of compaction of a Rhodic Eutrudox for four compaction levels: minimum tillage with chiseling (MTC), no-tillage (NT), no-tillage with four tractor passes (NTC4), and no-tillage with eight harvester passes (NTC8).

Depth	θs	θr	α	n	*K*_s_	BD	DC
cm	cm^3^ cm^−3^	cm^3^ cm^−3^	cm^−1^	–	cm day^−1^	g cm^−3^	%
	MTC						
0–10	0.585	0.198	0.1927	1.2691	83.78	1.10	71
11–20	0.553	0.200	0.1313	1.1839	83.78	1.16	76
21–30	0.526	0.200	0.0469	1.1469	57.26	1.27	83
31–40	0.550	0.200	0.0512	1.1654	35.70	1.16	76
41–50	0.554	0.198	0.0583	1.1679	44.70	1.10	71
	NT						
0–10	0.555	0.198	0.0892	1.1848	39.36	1.21	79
11–20	0.537	0.200	0.0822	1.1503	39.36	1.26	82
21–30	0.539	0.200	0.0756	1.1407	54.15	1.26	82
31–40	0.539	0.200	0.0756	1.1407	54.15	1.16	76
41–50	0.539	0.200	0.0756	1.1407	54.15	1.10	71
	NTC4						
0–10	0.508	0.200	0.0128	1.1782	26.09	1.35	88
11–20	0.510	0.200	0.0252	1.1391	26.09	1.34	87
21–30	0.524	0.197	0.0180	1.1494	26.98	1.32	86
31–40	0.550	0.200	0.0512	1.1654	35.70	1.16	76
41–50	0.554	0.198	0.0583	1.1679	44.70	1.10	71
	NTC8						
0–10	0.499	0.200	0.0017	1.2480	18.39	1.39	91
11–20	0.505	0.200	0.0102	1.1485	18.39	1.37	90
21–30	0.526	0.200	0.0211	1.1307	15.29	1.33	87
31–40	0.550	0.200	0.0512	1.1654	35.70	1.16	76
41–50	0.554	0.198	0.0583	1.1679	44.70	1.10	71

*θ*r, *θ*s, *α*, and n are van Genuchten’s parameters; K_s_: saturated hydraulic conductivity; BD, soil bulk density; DC, degree of compaction.

**Table 3:** Root architectural parameters of maize (*Zea mays*).

Symbol	Parameter name	Units	Values^1^ (mean, SD)
*Primary root*			
r_e_	Initial tip elongation rate	cm day^−1^	(2, 0)
a	Root radius	cm	(0.13, 0)
l_a_	Length of apical zone	cm	(0.15, 0)
l_b_	Length basal zone	cm	(0.15, 0)
l_n_	Internodal distance	cm	(0.88, 0)
n_b_	Maximum number of branches	–	(90, 0)
σ	Expected change of root tip heading	rad cm^−1^	0.2
Type	Type of tropism	–	1
N	Strength of tropism	–	1.5
dx	Spatial resolution along root axis	cm	0.25
*First-order laterals*			
r_e_	Initial tip elongation rate	cm day^−1^	(0.75, 0)
a	Root radius	cm	(0.05, 0)
θ	Insertion angle	rad	(1.2217, 0)
l_a_	Length of apical zone	cm	(3, 0)
l_b_	Length basal zone	cm	(2, 0)
l_n_	Internodal distance	cm	(0.89, 0)
n_b_	Maximum number of branches	–	(5, 0)
σ	Expected change of root tip heading	rad cm^−1^	0.3
Type	Type of tropism	–	1
N	Strength of tropism	–	1
dx	Spatial resolution along root axis	cm	0.25
*Second-order laterals*			
r_e_	Initial tip elongation rate	cm day^−1^	(0.75, 0)
a	Root radius	cm	(0.03, 0)
θ	Insertion angle	rad	(1.22173, 0)
k	Maximal root length	cm	(5, 0)
σ	Expected change of root tip heading	rad cm^−1^	0.4
Type	Type of tropism	–	1
N	Strength of tropism	–	0
dx	Spatial resolution along root axis	cm	0.25
*Basal or seminal roots*			
r_e_	Initial tip elongation rate	cm day^−1^	(3, 0)
a	Root radius	cm	(0.01, 0)
θ	Insertion angle	rad	(1.39626, 0)
l_a_	Length of apical zone	cm	(2, 0)
l_b_	Length basal zone	cm	(0.15, 0)
l_n_	Internodal distance	cm	(0.88, 0)
n_b_	Maximum number of branches	–	(90, 0)
σ	Expected change of root tip heading	rad cm^−1^	0.1
Type	Type of tropism	–	1
N	Strength of tropism	–	1.5
dx	Spatial resolution along root axis	cm	0.25
basal_first	First occurrence	days	3
basal_delay	Interim time	days	2
basal_max	Maximal number of basal roots	–	60
*Shoot-born or crown roots*			
r_e_	Initial tip elongation rate	cm day^−1^	(3, 0)
a	Root radius	cm	(0.01, 0)
θ	Insertion angle	rad	(1.5708, 0)
l_a_	Length of apical zone	cm	(2, 0)
l_b_	Length basal zone	cm	(2, 0)
l_n_	Internodal distance	cm	(0.88, 0)
n_b_	Maximum number of branches	–	(90, 0)
σ	Expected change of root tip heading	rad cm^−1^	0.05
Type	Type of tropism	–	1
N	Strength of tropism	–	1
dx	Spatial resolution along root axis	cm	0.25
sb_first	Emergence time of first shoot born root	days	1
sb_delay	Time delay between the emergence of shoot born roots	days	1.2
sb_nCR	Number of shoot born roots per root crown	–	11
sb_delayRC	Time delay between the emergence of root crowns	days	33
sb_dzRC	Distance between root crowns along the shoot	cm	1

^1^Root parameters values are adapted and calibrated from Leitner et al. (2010b).

The results of the model are variables of the soil (water balance, infiltration, runoff and deep drainage, real evaporation, water content, matric potential, soil mechanical resistance to penetration, and unsaturated hydraulic conductivity) and the crop (root system distribution, root length density, actual transpiration and water uptake) over time for each soil layer. Root length density was calculated for each depth for a single plant in an area of 0.12 m^2^ plant^−1^ (90 cm length and 14 cm width), *i.e.*, with plant density of 80,000 plants ha^−1^.

### Performance Evaluation of the Maize Root Growth Simulation Model

The agreement between the measured and simulated values was expressed by the mean absolute error (Casaroli et al., 2010), the root-mean-square error (de Jong van Lier et al., 2008), and the coefficient of residual mass (Bonfante et al., 2010). The precision was determined by the correlation coefficient (*r*) (Addiscott and Whitmore, 1987) and the accuracy by means of the Willmott concordance index (*d*) (Willmott et al., 2012), while the evaluation of modeling performance was made using the efficiency of the model (EF) (Bonfante et al., 2010) and the proximity of the 1:1 ratio.

### Data Analysis

The results for grain yield, dry shoot yield, root length density, and root dry mass were subjected to ANOVA (*p*<0.05), and when the effects of the treatments were significant, the means were compared by Tukey test at a 5% error probability level. The analyses were performed using the Statistical Analysis System (SAS) 8.0 software.

## Results

**Figure 2:**
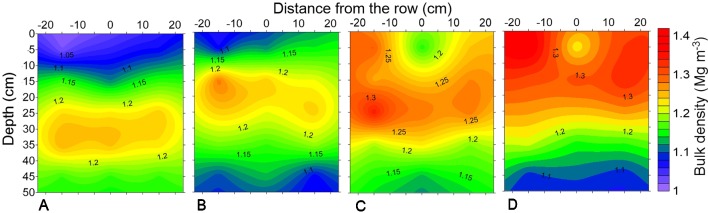
Soil bulk density of a profile of Rhodic Eutrudox under **(A)** minimum tillage with chiseling, **(B)** no-tillage, **(C)** no-tillage with four tractor passes, and **(D)** no-tillage with eight harvester passes.

The horizontal and vertical variability of soil density in February 2014 can be observed in **Figure 2**. The compaction levels after soybean cultivation demonstrated the persistence of the differences before the sowing of the second maize crop. Soybean cultivation in the 2013/2014 cropping season, prior to maize cultivation, in association with the effects of the shank-type furrow opener favored reductions in bulk density in the sowing row, mainly in the trafficked areas (**Figures 2C, D**), which was important for improved maize root depth in the following crop. The effects of tractor and harvester traffic could be observed up to 30 cm deep, while chiseling had a residual effect up to 20 cm.

**Figure 3:**
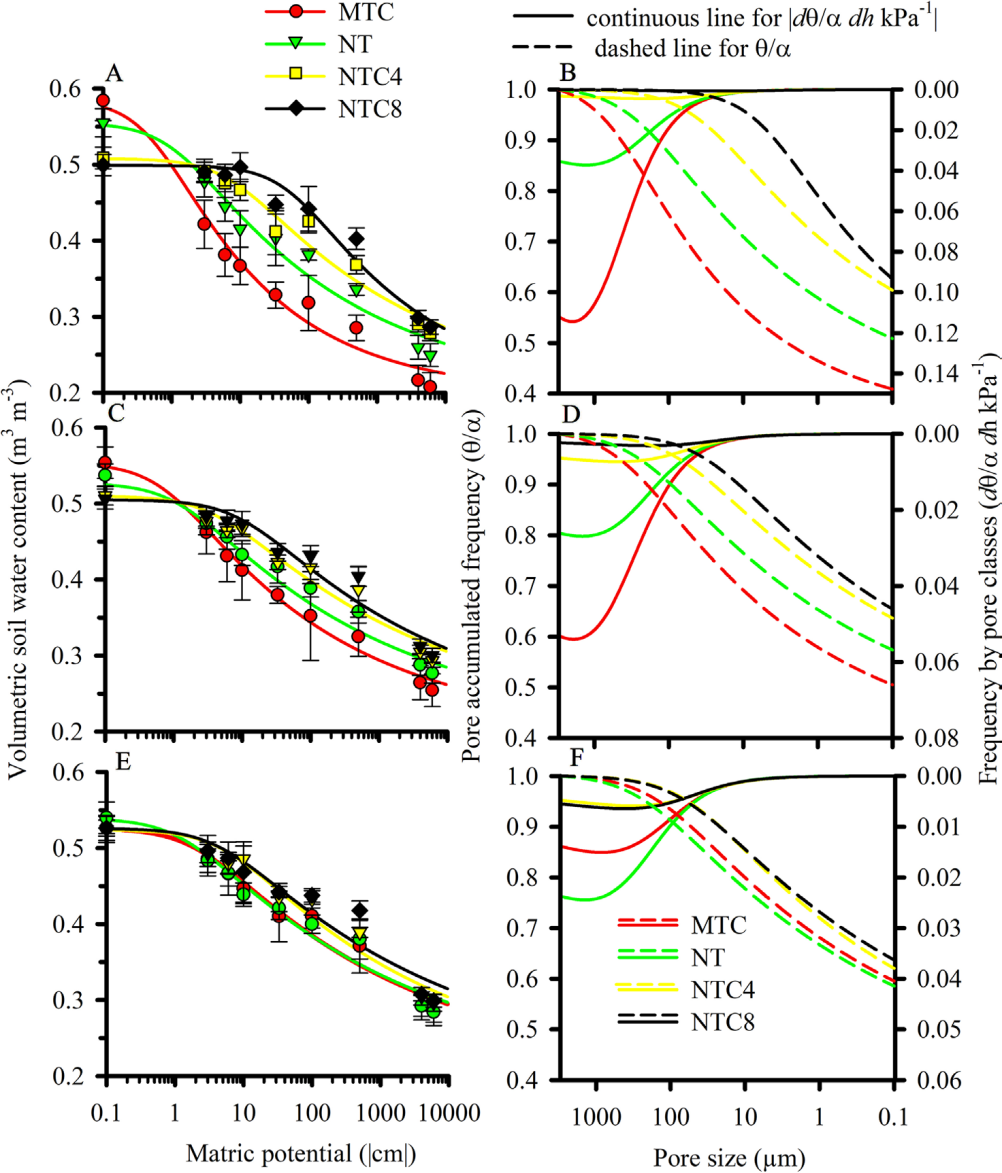
Water retention curve **(A, C, E)** and cumulated frequency and distribution of pore size **(B, D, F)** in the 0–10 cm **(A, B)**, 10–20 cm **(C, D)**, and 20–30 cm **(E, F)** layers due to the compaction level of a Rhodic Eutrudox. MTC: minimum tillage with chiseling; NT: no-till system; NTC4: no-tillage with four passes of a tractor; NTC8: no-tillage with eight passes of a harvester. Bars indicate the SD.

**Figure 4:**
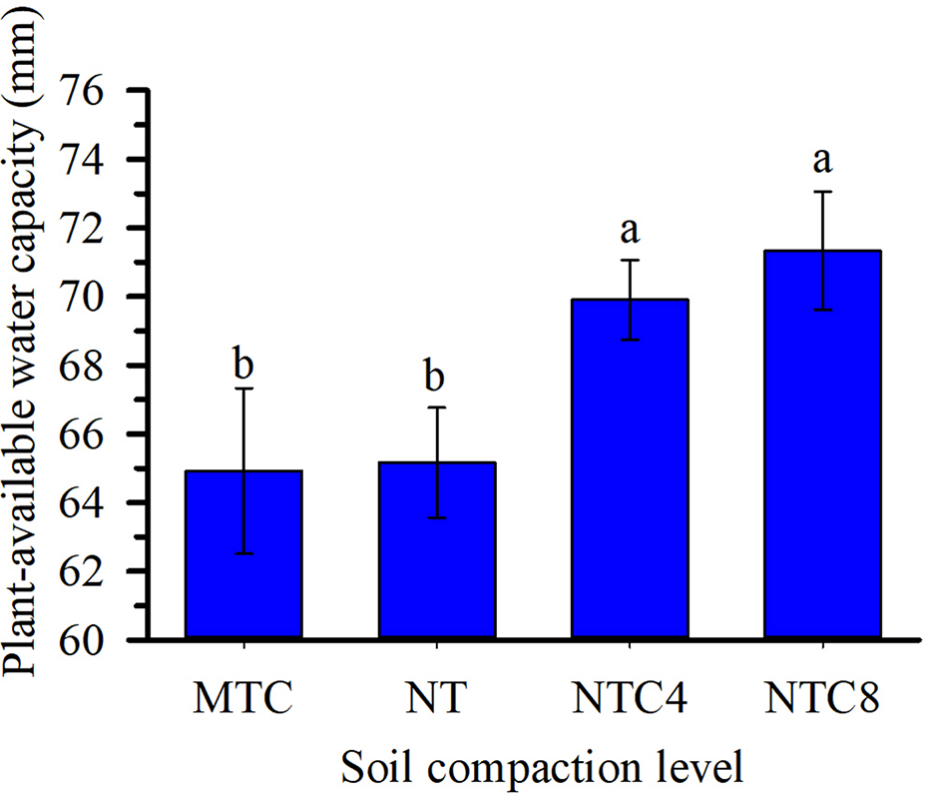
Plant-available water capacity in the 0–50 cm depth under soil compaction levels of a Rhodic Eutrudox. Means followed by the same letters do not differ by Tukey’s test at the 5% level; Bars indicate the standard error of the mean. MTC: minimum tillage with chiseling; NT: no-till system; NTC4: no-tillage with four passes of a tractor; NTC8: no-tillage with eight passes of a harvester.

The soil water retention curves were altered due to the soil compaction level (**Figure 3**). Agricultural traffic reduced the total soil porosity, thus altering the frequency and distribution of the pores in the soil (**Figures 3B, D, F**), and the values of the corresponding soil hydraulic parameters (α and *n*) of the soil water retention curves (**Table 2**). Because of agricultural traffic, the water retention was altered, requiring higher tensions for the extraction of water from the soil. For the same matric potentials, between field capacity (−10 kPa) and the permanent wilting point (−1,500 kPa), increases were observed in the water contents due to the increase in the soil compaction level (**Figure 3**), increasing the plant-available water capacity in compacted soil (**Figure 4**). The effects of the traffic with tractor or harvester in the no-till system presented increases in soil water retention for all of the layers up to 30 cm in depth, while soil chiseling provided reductions in water retention, principally up to 20 cm in depth (**Figure 3**).

**Figure 5:**
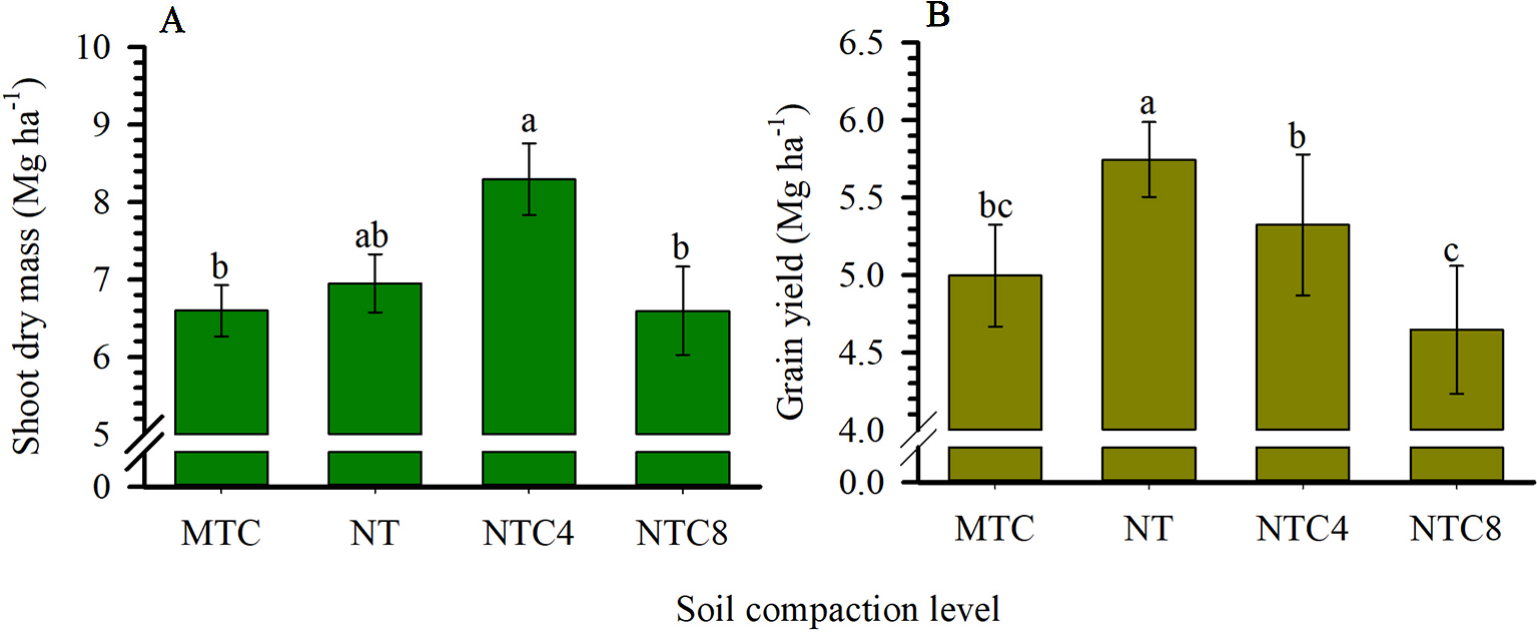
Dry mass of the shoot of the plant **(A)** and grain yield **(B)** of maize grown in different compaction levels of a Rhodic Eutrudox. MTC: minimum tillage with chiseling; NT: no-till system; NTC4: no-tillage with four passes of a tractor; NTC8: no-tillage with eight passes of a harvester. Means followed by the same letters do not differ by Tukey’s test at the 5% level. Bars indicate the standard error of the mean.

The dry mass of the shoot of the plant was greater in the no-tillage system and in the soil subjected to tractor traffic (**Figure 5A**). The increase in compaction by traffic with the harvester or the chiseling of the soil caused reductions in the biomass production of the shoot compared to the soil trafficked with the tractor. Maize grain yield was reduced in the chiseled area and in areas with additional compaction by tractor and harvester traffic compared to the soil under the no-tillage system (**Figure 5B**).

**Figure 6:**
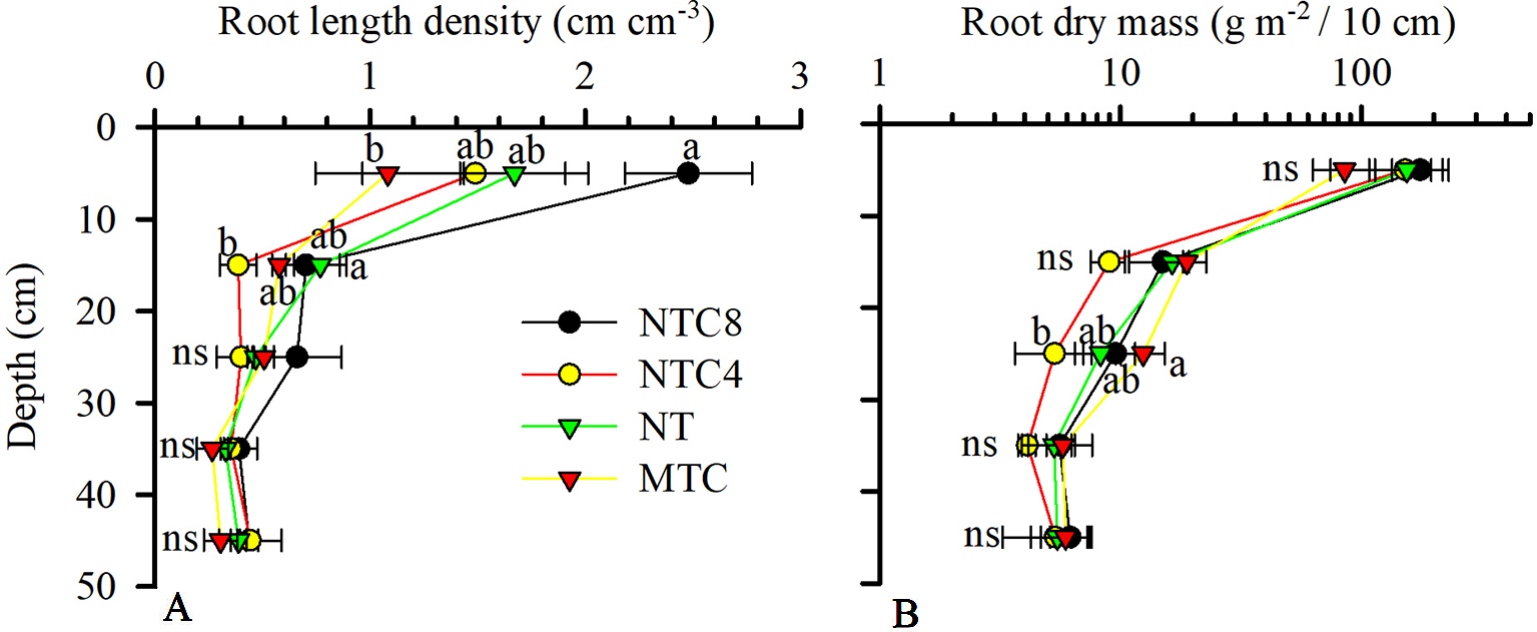
Maize root length density **(A)** and dry mass (log scale) **(B)** in 1D in the soil profile because of compaction levels in a Rhodic Eutrudox. MTC: minimum tillage with chiseling; NT: no-till system; NTC4: no-tillage with four passes of a tractor; NTC8: no-tillage with eight passes of a harvester; ns: not significant. Means followed by the same letters do not differ by Tukey’s test at the 5% level. Bars indicate the SEM.

**Figure 7:**
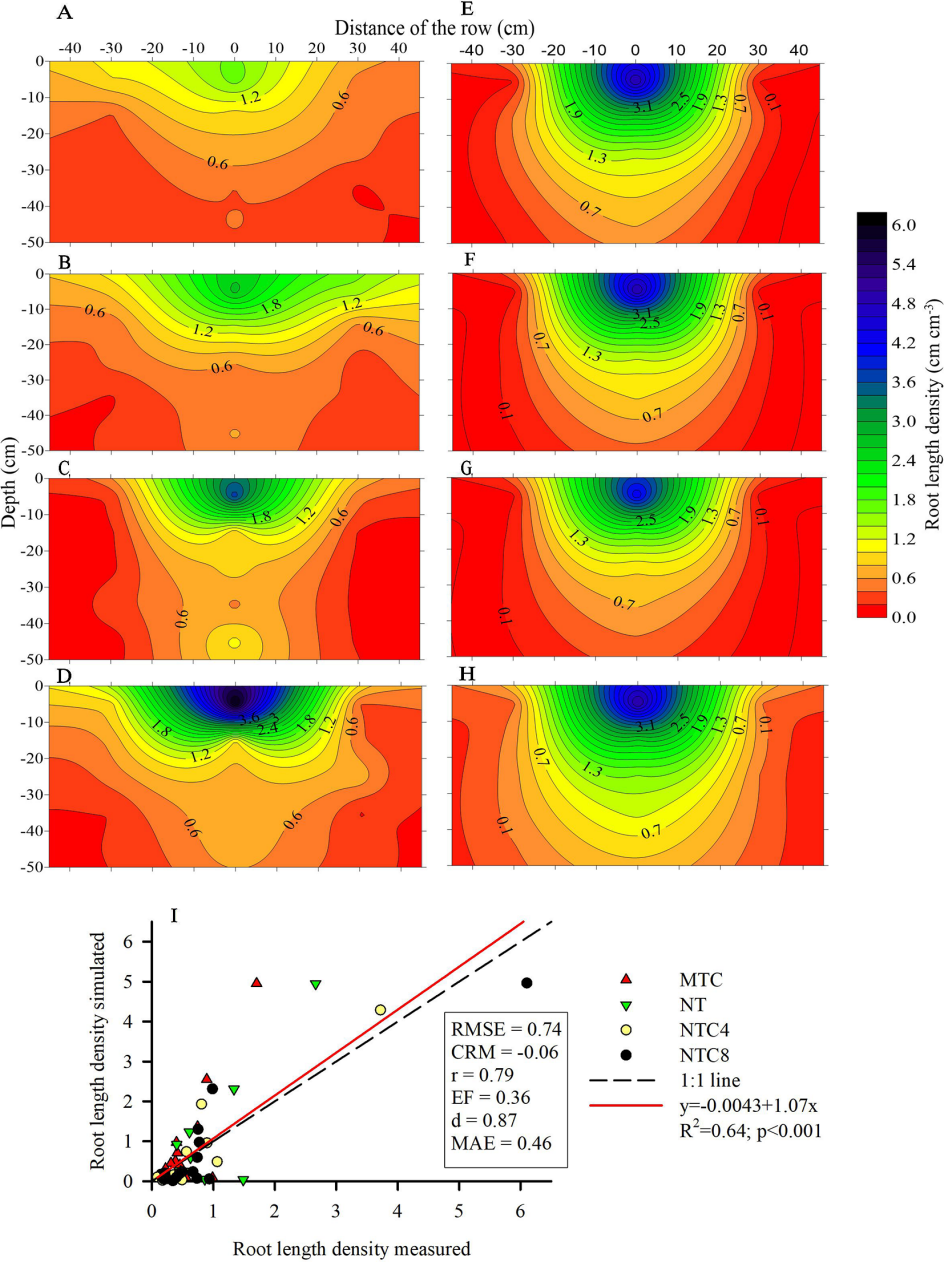
Root length density of maize (*Zea mays*) measured at field condition **(A–D)** and modeled **(E–H)** in 2D in soil profile under four soil compactions levels: **(A, E)** reduced tillage system (MTC), **(B, F)** no-tillage system (NT), **(C**, Results no-tillage system with four tractor passes (NTC4), or **(D, H)** no-tillage system with eight harvester passes (NTC8), and (I) relationship of simulated and measured root length density in a Rhodic Eutrudox.

The growth of the root system of the maize crop was altered due to the soil compaction level (**Figures 6** and **7**). The root length density (**Figure 6A**) and the root dry biomass (**Figure 6B**) indicated changes in soil profile due to traffic or soil chiseling. The root length density was altered up to 20 cm deep (**Figures 5** and **6**), while for the root dry mass there were changes in the 20–30 cm layer. The chiseling of the soil provided increases in the root dry biomass of maize in the 20–30 cm layer in relation to the tractor trafficked soil. In the 10–20 cm layer, there were reductions in root length in the soil trafficked with the tractor in relation to the no-tillage system. However, in the surface layer (0–10 cm), the soil chiseling reduced root length density in relation to the soil trafficked with the harvester (**Figure 6**).

The 2D root distribution in the soil profile in relation to the row and interrow of the maize crop was altered due to the soil compaction level (**Figure 7**). The increase in the compaction levels in the soil favored increases in the quantity of roots in the soil profile (**Figures 7C, D**) in relation to the chiseled soil (**Figure 7A**). Principally in the 0–10 cm layer, the harvester traffic (NTC8) provided increases in the root length density in relation to the chiseled soil (**Figure 6A**). This increase in root length density in NTC8 was conditioned by the higher root concentration near the maize sowing line (**Figure 7D**).

**Figure 8:**
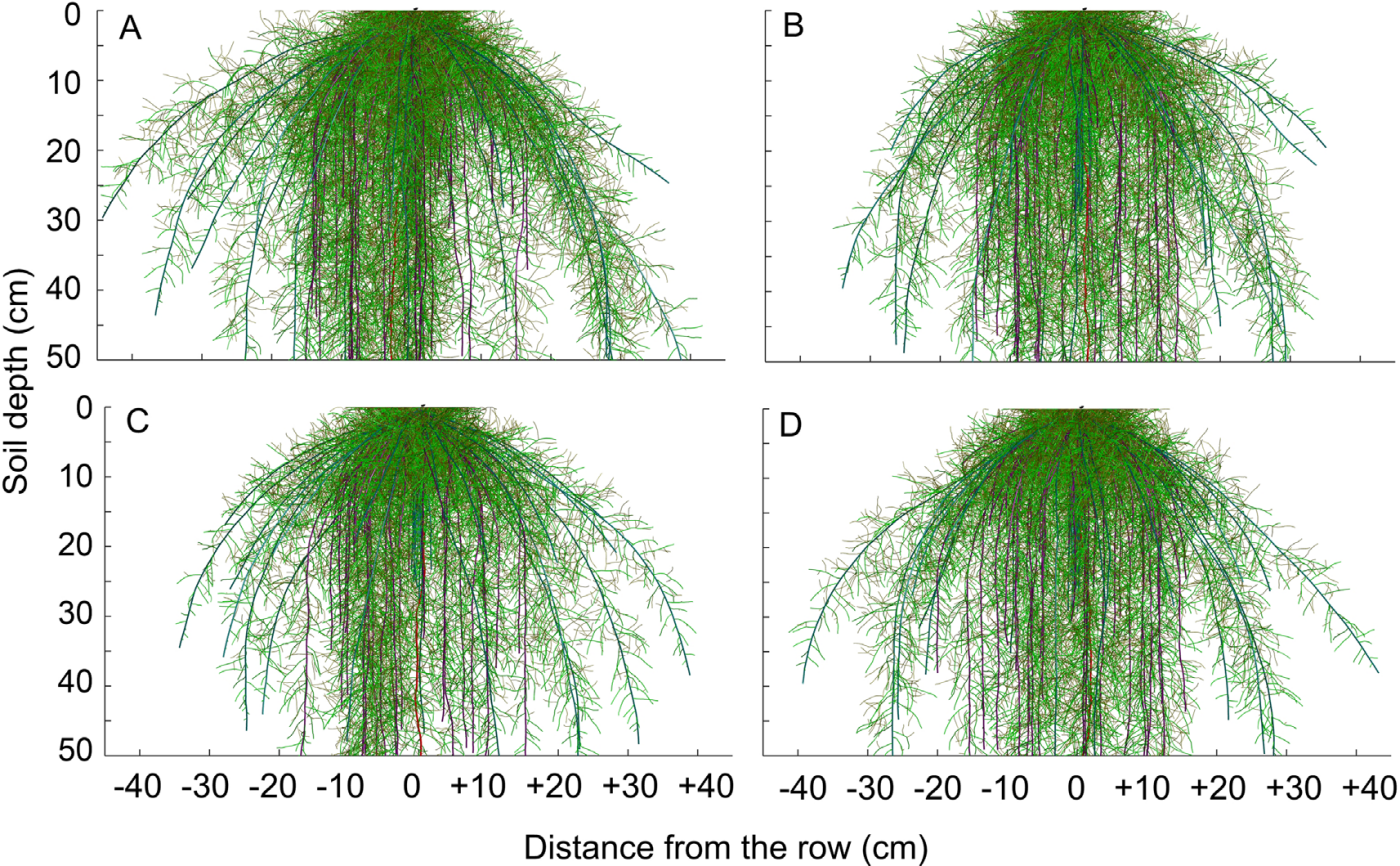
Visualization of simulated root system architecture in 3D soil profile under **(A)** reduced tillage system, **(B)** no-tillage system, **(C)** no-tillage system with four tractor passes, or **(D)** no-tillage system with eight harvester passes in a Rhodic Eutrudox.

The modeling of the root growth for the maize crop was performed for the development period between sowing and root sampling, which corresponded to 110 days (**Figure 8**). The architecture of the maize root system was altered by the compaction level, regarding the number and the length of the roots.

**Figure 9:**
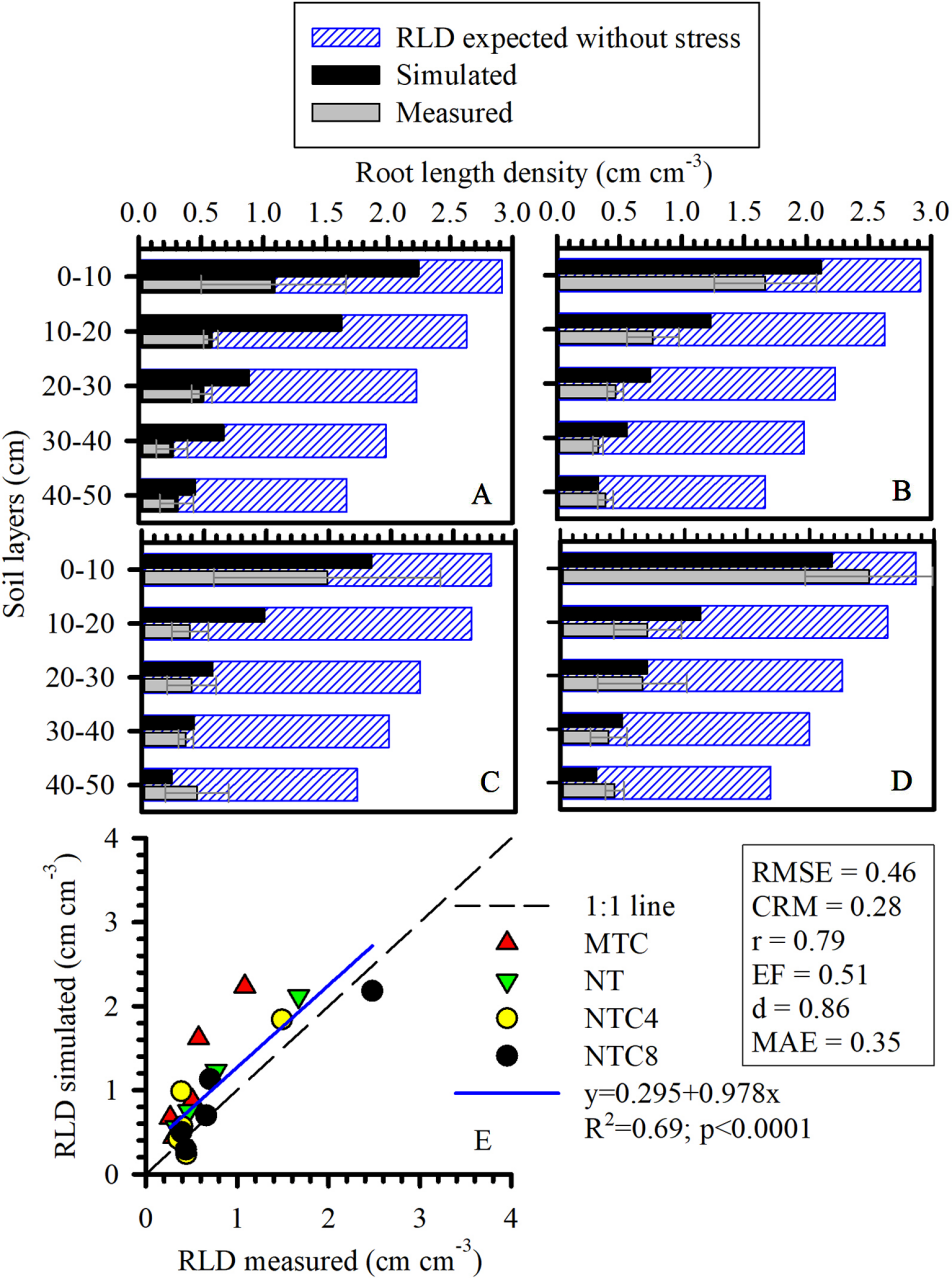
One-dimensional distribution (1D) of root length density (RLD) of maize simulated, measured, and expected without stress under **(A)** reduced tillage system (MTC), **(B)** no-tillage system (NT), **(C)** no-tillage system with four tractor passes (NTC4), or **(D)** no-tillage system with eight harvester passes (NTC8), and **(E)** relationship of simulated and measured RLD in a Rhodic Eutrudox. RMSE: root-mean-square error; CRM: coefficient of residual mass; MAE, mean absolute error.

Physical limitations were incorporated into the root growth model. From the 3D explicit root architecture as shown in **Figure 8**, root length density profiles were computed (**Figures 7E–H**) and compared to the values that had been measured in the field (**Figure 9**). In addition, measured root length densities in 2D agreed with simulated values of root length density (**Figure 7I**). The efficiency of the model in 2D equaled to 0.36, compared to 0.51 in 1D. Among the compaction levels, we observed an overestimate in the reduced tillage system of the root length density in relation to the values measured in the field (**Figure 9A**). In the other compaction levels, the simulated and measured values were similar. In all treatments, there were reductions of root growth in relation to the potential of root growth without physical stress (**Figure 9**). Thus, the maximum reduction of root growth at field in relation to a condition modeled without physical stress was reached in the MTC (76%). In addition, simulated root growth in soil profiles considering soil physical condition (stress reduction function) improved the results regarding root length density, and therefore this approach should be used in simulation scenarios that consider water uptake. The relationship between measured and simulated root length density values in 1D (**Figure 9E**) indicates that the simulated values agreed with the values observed in the field. The Camargo index (*d*) of 0.86, which evaluates the agreement of simulated and measured values, indicated that the model was adequate for predicting the maize root growth observed in the field.

**Figure 10:**
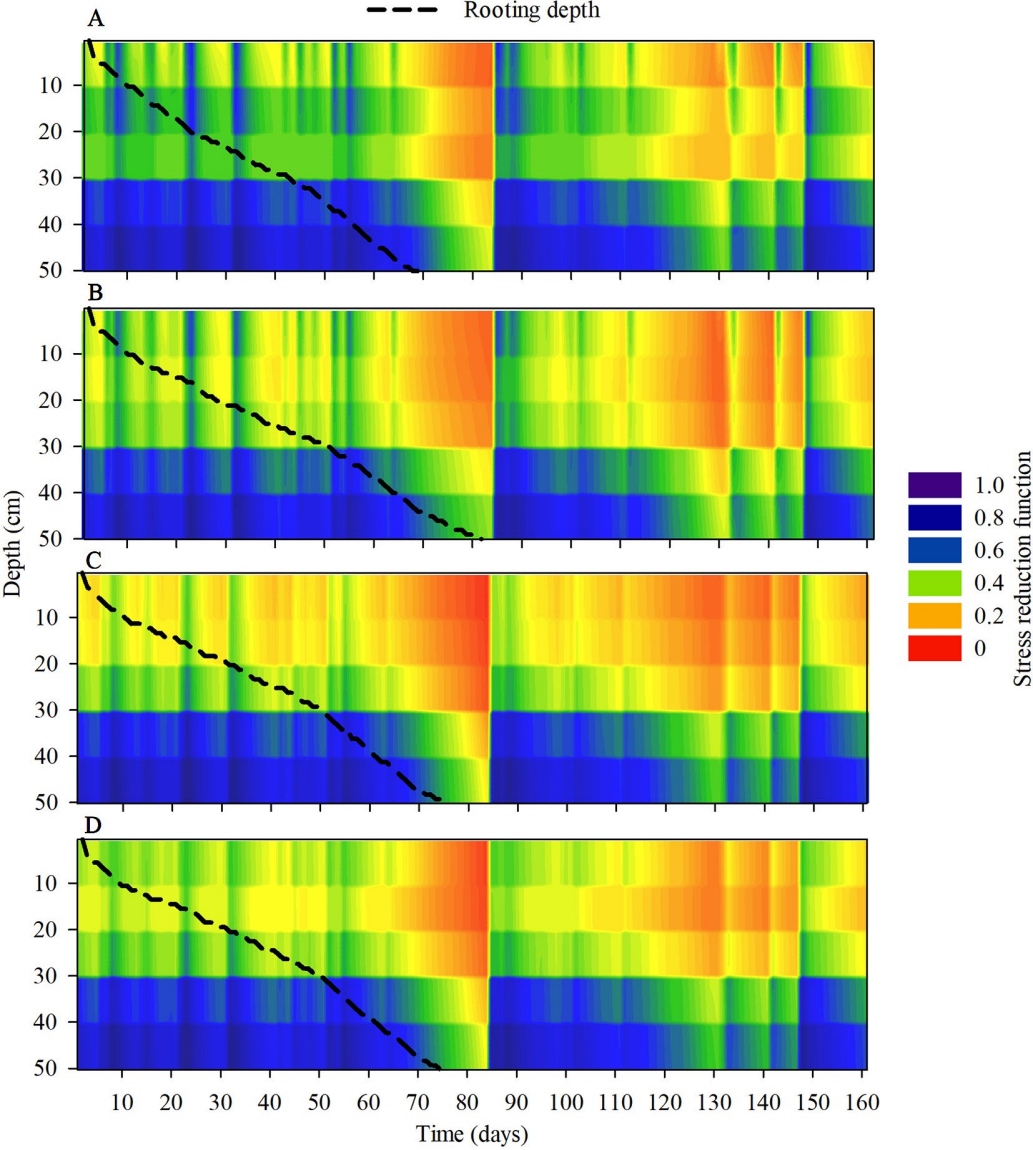
Modeling the dynamics of the stress reduction function for root elongation and root system depth (dotted lines) under **(A)** reduced tillage system, **(B)** no-tillage system, **(C)** no-tillage system with four tractor passes, or **(D)** no-tillage system with eight harvester passes in a Rhodic Eutrudox.

**Figure 11:**
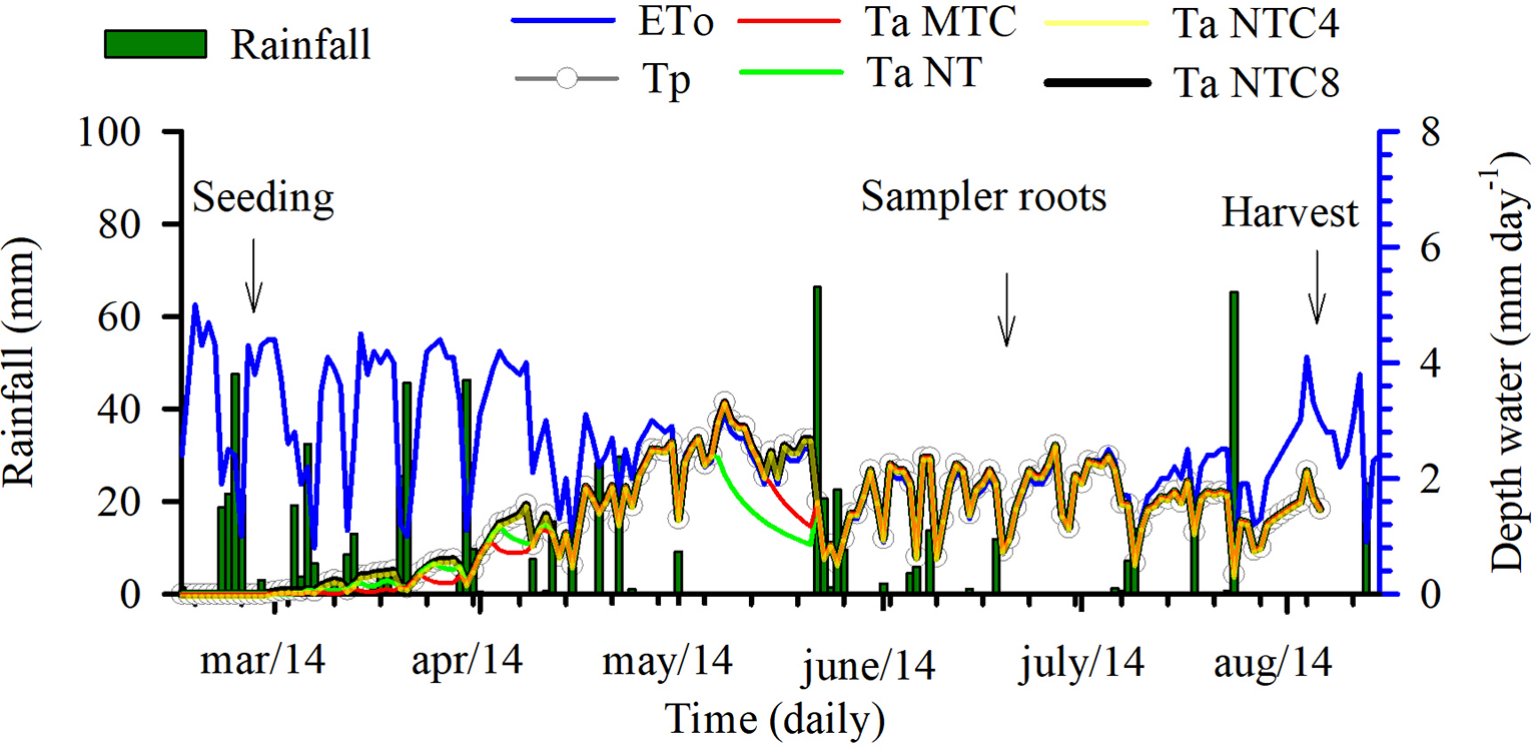
Weather data of precipitation, potential evapotranspiration (ETo), and potential (Tp) and current (Ta) transpiration for four levels of compaction in a Rhodic Eutrudox. MTC: minimum tillage with chiseling; NT: no-till system; NTC4: no-tillage with four passes of a tractor; NTC8: no-tillage with eight passes of a harvester.

The modeling of the stresses of root elongation and root growth during the maize development cycle indicated that there were changes in the soil physical conditions during the crop cycle under the compaction levels (**Figure 10**). The transpiration rate of maize, during the initial period of development, was lower in the areas with chiseled soil than in the other treatments (**Figure 11**). The depth of the root system and the root elongation rate were altered due to the water and mechanical stress in the soil profile over a growth season. The time required for the roots to reach at least 50 cm in the soil profile was altered due to the soil compaction level; in the reduced tillage system, this occurred at least 10 days earlier than in the no-till areas (**Figure 10**). This occurred because of the greater rate of root elongation under conditions of lower soil physical limitation.

**Figure 12:**
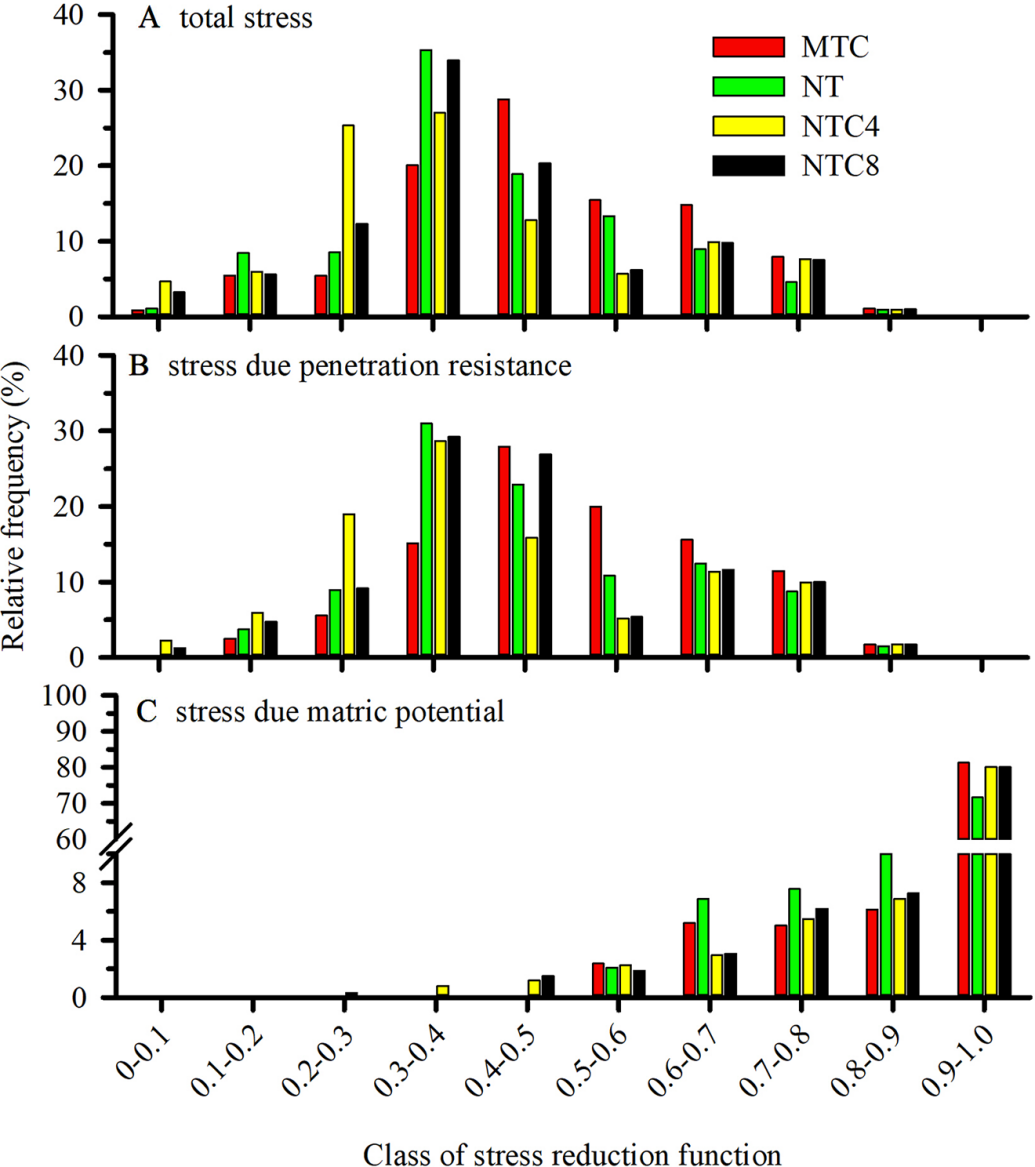
Frequency of the total stress **(A)** or soil resistance to penetration **(B)** and matric potential **(C)** for root elongation during the maize development cycle in a Rhodic Eutrudox. The x-axis represents classes of stress reduction function. Values close to zero correspond to the absence of growth, while values close to 1 mean maximum potential of root growth. MTC: reduced tillage system with soil chiseling; NT: no-till system; NTC4: no-tillage with four passes with a tractor; NTC8: no-tillage with eight passes with a harvester.

The rate of root elongation was reduced because of the combination of the stresses attributable to the soil resistance to penetration (**Figure 12B**) and to the matric potential (**Figure 12C**), which incorporates the effects of excess water (anoxia or hypoxia) or water deficiency. The contribution of the mechanical and hydric stresses on the total stress demonstrated that most of the stresses that caused root growth reduction were determined by mechanical stresses (**Figure 12B**). Thus, in areas with tractor or harvester traffic, there were greater contributions from mechanical stresses than from the hydric stresses (**Figure 12C**). In the MTC, the contribution of the hydric stresses presented greater weight and favored the reduction of the root elongation rate through the interaction of the water and mechanical stresses.

Thus, the reduction of the overall values of root elongation (**Figure 12A**) should be interpreted as daily effects for each root at each position in the soil (**Figure 10**). The frequency and intensity of the stresses for root elongation describe the physical conditions to which the maize roots were subjected during the entire development period of the crop. The soil compaction levels caused changes in the physical condition of the soil resistance to penetration or the matric potential for the root elongation. For most of the crop development period, stress caused by soil resistance to penetration (**Figure 12B**) was the principal reason for the reduction of maize root growth. In some periods, the reduction of the water content in the soil provided increases in hydric stress, and this caused reductions in the stress parameter for root growth.

## Discussion

Hydric and mechanical stress of maize root elongation was successfully incorporated into the root growth model in 3D, into the RootBox (Leitner et al., 2010a; Moraes et al., 2018a). The association of the root growth model with the flow of water in 1D (van Dam and Feddes, 2000) and the absorption of water by the roots due to the matric flux potential (de Jong van Lier et al., 2008) represent an advancement in the understanding of biophysical processes in the soils. The model was able to predict maize root growth due to soil physical conditions (**Figure 8**). In the future, this model could be associated with other models to predict root growth based on climatic predictions and allow the anticipation of agricultural strategies to prevent limiting conditions to root growth from arising in agricultural crops.

The soil compaction, caused by the harvester traffic, resulted in significant reductions in grain yield and shoot biomass (**Figure 5**). However, the soil chiseling also reduced these parameters in relation to the no-tillage system (**Figure 5**). The harvester traffic provided increases in the number of roots in the soil profile, principally in the regions close to the sowing furrow (**Figure 7**). In general, there were changes in the soil profile in the distribution of the root system, mainly in the 30 cm nearest to the sowing row.

Soil compaction levels increased the physical limitations to root growth, as was described within the model by the frequency of the total stresses (**Figure 12A**). Our simulations enable a quantified hypothesis whether this stress is due to soil penetration resistance (**Figure 12B**) or due to the effect of the water matric potential in the soil (**Figure 12C**). Therefore, the model offers a way to analyze and explain how hydric and mechanical stresses during the development cycle are altered due to the meteorological conditions precipitation and evapotranspiration (**Figure 11**). This is of high relevance since these factors are responsible for changes in the soil physical limitation to maize root elongation (**Figure 10**).

The intermediate levels of bulk density, in the no-tillage system, provided the best physical conditions for maize crop production performance (**Figure 5**), as was described due to soil physical quality in a long-term tillage system (Moraes et al., 2016). Thus, soil structure and its physical quality was damaged due to soil chiseling, reducing the plant-available water capacity in chiseling soil (**Figure 5**) as it was with soil compaction, with increasing of mechanical impedance, and they both reduced the grain yield of maize. In trafficked soil, there was an increase in plant-available water capacity. That happened due to changes in soil retention curve with higher reductions of macropores but few changes in micropores in compacted soils. Thus, the water storage in the soil was affected by changes in pore size distribution, which decrease larger pores and increase micropores promoting increases in water volume storage in soil profile. Therefore, reduction of macropores in compacted soil affected soil aeration, which affects root growth and grain yield. In a long-term experiment in an Oxisol soil, chiseling reduced the yield grain of soybean (Moraes et al., 2017b). Thus, in loose soil, the main factor to reduce the yield and root length density should be due to the reduction of plant-available water capacity (**Figure 4**) promoted by reduction of size and stability of soil aggregates (Nunes et al., 2015) in chiseled areas. Thus, there was an increase in the water stress to crop growth (**Figures 10A** and **12C**) in chiseled soils.

The changes in the architecture of the maize root system (**Figure 8**) in the soil profile (**Figure 7**) were the result of the physical-hydric stress levels during the development cycle (**Figure 10**). The increase in the root length under the highest degree of compaction was associated with pore connectivity in the soil profile (Bengough, 2012a) which promotes reduction in root diameter and increases of root length (Duruoha et al., 2007). The increase in root length density, mainly in the 0–10 cm layer (**Figure 6**), is principally due to the confinement of the roots in the sowing furrow (**Figure 7**), where there were better physical conditions for root growth. This confinement of the roots due to increasing compaction levels directed the roots to sub-superficial layers (∼30 cm depth) nearest to the sowing line (**Figure 7**). Thus, this root system confinement in compacted soil reduced the soil volume explored in superficial soil layers and increased the volume in deeper soil layers. This affected water uptake by the root system, as previously observed in structured subsoil by White and Kirkegaard (2010). In addition, the soil type and management must be considered and are likely to be more important factors than genotype to promote increased rooting depth (Gao et al., 2016).

The modeling was efficient at predicting the maize root growth (**Figure 8**) observed in the field (**Figure 7**) in 1D (**Figure 9E**) at the soil profile, and was useful for observing the effects of hydric and mechanical stress during cultivation. However, root system length distributions in 2D in the field was not very well simulated by the model (**Figure 7I**), especially for loose soil (MTC). Materechera et al. (1991) showed in a study with many monocotyledons and dicotyledonous that root elongation of maize in compacted soil (0.44 mm day^−1^) was higher than all other cereals (barley 0.31 mm day^−1^; oats 0.32 mm day^−1^; rice 0.31 mm day^−1^; sorghum 0.34 mm day^−1^; wheat 0.41 mm day^−1^). This higher penetration of maize roots was due to an increased root diameter (maize with 1.39 mm day^−1^) compared with other cereals (barley 0.066 mm day^−1^; oats 0.076 mm day^−1^; rice 0.056 mm day^−1^; sorghum 0.078 mm day^−1^; wheat 0.076 mm day^−1^). Roots of maize grew slower than dicotyledonous in compacted soil (*e.g.*, lupine 0.71 mm day^−1^, pea 0.70 mm day^−1^, faba bean 0.68 mm day^−1^, soybean 0.57 mm day^−1^, and cotton 0.45 mm day^−1^). Materechera et al. (1991) suggest that there was a significant positive correlation (*r*=0.78, *p*<0.05) between root diameter and elongation over all the species in stressed plants. In addition, the study showed that dicotyledonous species were more able to elongate in the strong medium than monocotyledons due to the higher root diameter of dicotyledonous. Thus, the higher diameter of crown roots of maize can facilitate root penetration in harder soils better compared to other cereals (Materechera et al., 1991). This indicates that grasses experience better developmental conditions under soil compaction, principally because of the adventitious seminal roots that are emitted from the crowns of the stem above the surface of the soil. In addition, in a dicotyledonous architecture, the taproot, which determines the growth direction of root system, is more affected by soil physical limitations that can alter the root direction in the soil profile. However, there was an overestimate of the root length density in loose soil (MTC) (**Figure 9**), probably because the water stress was greater in field than that simulated in the model. This discrepancy should be due to soil structure changes due to soil chiseling (Moraes et al., 2016), which affect the storage (**Figure 4**) and flux of the water in the soil profile, and this is not reflected well in the soil water retention curve (**Figure 3**). In addition, there are other factors (*e.g.*, hydraulic root resistance, root radial and axial conductivity, root mucilage and rhizosphere hydraulic properties) that were not included in this model, as well as biological (*e.g.*, rhizosphere microorganisms) and chemical factors (*e.g.*, chemical signals, redox potential, and nutrient available) (Vereecken et al., 2016) that could affect the root growth.

Models of root growth, because of soil hydric and mechanical stress on the elongation of roots, are dynamic in time and space and are, therefore, useful for the interpretation of the soil alterations that can affect plant growth. Mechanical stress for root elongation can be changed due to soil structure (*e.g.*, presence of biopore and pore continuity) (Bengough, 2012a). Thus, in this root growth model, the mechanical stress to root elongation is represented by the soil penetration resistance curve, which can be used to estimate the relation between root resistance and penetrometer resistance (Bengough and Mullins, 1991). Also, an age-hardening effect of soil tillage over time can be included into the model by modifying the soil penetration resistance curve (Moraes et al., 2017a). These studies with root growth modeling (*i.e.*, different root systems) (Tron et al., 2015) can help to understand the root contribution to produce biopores in the soil profile, especially in areas under no-tillage. The root system of maize crop was influenced only little by soil compaction, presenting high resilience to overcome the problems caused by the agricultural traffic in this Rhodic Eutrudox. In addition, maize root growth was not limited by compaction due to machine traffic in an Ultisol, and there were increments of grain yield due to increment of soil water retention in sand soils with traffic (Moraes et al., 2018b); this indicates that maize could be an option to use in compacted soils.

Observed root length density profiles follow an exponential decay shape as commonly known for maize (Pagès et al., 2004). They reach a depth up to 2 m (Canadell et al., 1996), and the highest root length density was observed near the sowing furrows in the soil trafficked by the harvester (**Figure 7D**), with values up to 6 cm cm^−3^. These values are close to the maximum root densities observed in the literature (Bengough, 2012b). Because the physical properties that limit root elongation (soil resistance to penetration, aeration, and water content) are heterogeneous and dynamic in time, the only way to infer their effect on overall root development and root water uptake was through modeling (**Figure 10**).

In line with results of, *e.g.*, Nosalewicz and Lipiec (2014), actual transpiration (*i.e.*, root water uptake) (**Figure 10**) was reduced with increasing soil compaction. Biopores decrease the axial pressure on the root tip, while still exerting radial pressures on the root in narrow pores (Bengough, 2012a) decreasing the root diameter (Jin et al., 2013). In the chiseled soil, which has a higher macropore density (Moraes et al., 2016), maize plants experienced greater periods of hydric stress. Continuous pores and biopores in soil profile improved water availability to plants, but macropores in chiseling soil are not very well connected in the soil profile (Pires et al., 2017). Thus, macropores in chiseled soil do not help to improve rooting depth due to changes in the soil water retention (Rabot et al., 2018) with faster water drainage (Armindo and Wendroth, 2016) and reduced unsaturated hydraulic conductivity (Ghanbarian-Alavijeh and Hunt, 2012).

As a result of the alteration of the soil structure by chiseling (Moraes et al., 2016), the roots of the crops were more susceptible to periods with hydric stress, increasing the frequency of days with greater reductions in root elongation due to the soil water matric potential (**Figure 12**). As a result, reductions in shoot biomass production (**Figure 5A**), grain yield (**Figure 5B**), and root length density (**Figures 6A** and **7A**) occurred in relation to the soil under no-tillage system.

## Conclusions

Maize root growth modeling, including soil physical limitations (mechanical and hydric stress), can be used for the analysis of architecture development associated with soil water flow models, increasing the fundamental understanding of stress that acts on the growing roots. Experimental data showed that the no-tillage system provided the best soil physical conditions for maximum maize yields. In the other scenarios, yield was reduced due to soil chiseling and/or through the excessive level of compaction caused by harvester traffic. Our simulations and analysis showed that the contribution of the mechanical stresses to the reduction of maize root growth was more important in areas with agricultural traffic; however, in areas with soil chiseling, the hydric stresses were the most important component of the total stresses on root elongation of maize, due to the increase of pore diameter and reduction of soil water retention. The root system of maize crop showed the potential of root length density increases due to increased soil compaction. The hypothesis of this work was confirmed that maize root growth could be simulated including mechanical and hydric stresses in the RootBox model. Our results improve the understanding of root growth in soils with physical limitations. As such, they facilitate the application of benchmarking models of root system development and water uptake. We showed that compared to the baseline scenario, explicit consideration of mechanical and hydric stresses improved the agreement between model and data.

## Author Contribution

MM, HD, and JF designed the field experiment and performed data analyses. MM and JB performed the field sampling and analyzed the soil and roots. DL and AS performed the root growth code. MM performed the root growth modeling. MM wrote the first draft of the manuscript. All authors contributed to manuscript revision, read and approved the submitted version.

## Conflict of Interest

The authors declare that the research was conducted in the absence of any commercial or financial relationships that could be construed as a potential conflict of interest.
