# Supplementary figures and images for: Mechanical and Hydric Stress Effects on Maize Root System Development at Different Soil Compaction Levels
Source: Front Plant Sci. 2019 Oct 29;10:1358. doi: 10.3389/fpls.2019.01358 (PMC6833975; doi:10.3389/fpls.2019.01358)

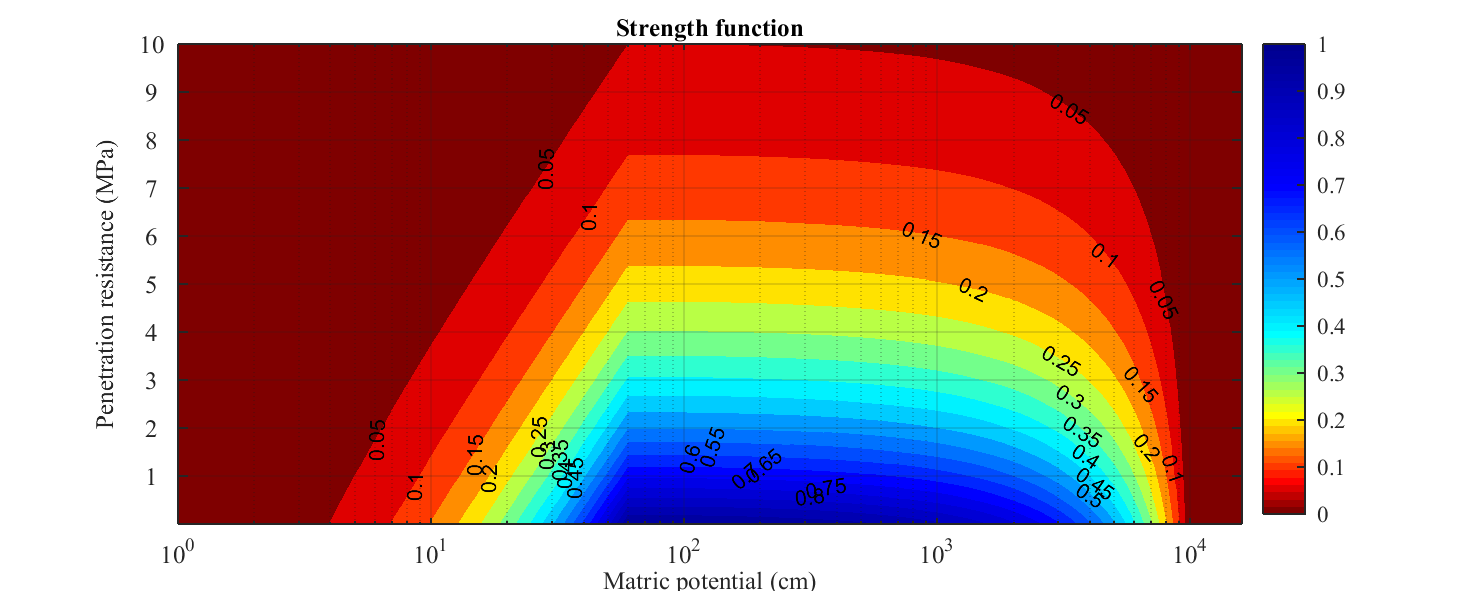

Supplement: Supplementary file 2 [file DataSheet_1.zip › Code_RootBox_MaizePaper_MoacirTuzzindeMoraes_Frontiers/strength_function.png]
